# Analysis of the *Phialocephala subalpina* Transcriptome during Colonization of Its Host Plant *Picea abies*

**DOI:** 10.1371/journal.pone.0150591

**Published:** 2016-03-08

**Authors:** Vanessa Reininger, Markus Schlegel

**Affiliations:** ETH Zurich, Institute of Integrative Biology, Universitätstrasse 16, 8092, Zurich; University of Innsbruck, AUSTRIA

## Abstract

**Background:**

*Phialocephala subalpina* belongs to the *Phialocephala fortinii* s.l.–*Acepphala applanata* species complex (PAC) forming one of the major groups belonging to the dark septate endophytes (DSE). Depending on the strain, PAC was shown to form neutral to pathogenic associations with its host plant *Picea abies*. To understand PACs lifestyle we investigated the effect of presence/absence of *Picea abies* on the transcriptome of strain 6_70_1.

**Materials and Methods:**

PAC strain 6_70_1 was grown in liquid Pachlewski media either induced by its host plant *Picea abies* or without host plant as a control. Mycelia were harvested in a time course (1, 2, 3, 4, 7, 11, 18 days) with and without induction by the host plant and the fungal transcriptome revealed by Illumina sequencing. Differential gene expression analysis over the time course comparing control and treatment at each time point using the ‘edgeR glm approach’ and a gene enrichment analysis using GO categories were performed.

**Results:**

The three main functional groups within differentially expressed genes were ‘metabolism’, ‘transport’ and ‘cell rescue, defense and virulence’. Additionally, genes especially involved in iron metabolism could be detected by gene set enrichment analysis.

**Conclusion:**

In conclusion, we found PAC strain 6_70_1 to be metabolically very active during colonization of its host plant *Picea abies*. A major shift in functional groups over the time course of this experiment could not be observed but GO categories which were found to be enriched showed different emphasis depending in the day post induction.

## Introduction

Fungal endophytes are a very diverse group colonizing different plant organs [1–3] and were shown to have several ecological functions such as increasing plants’ tolerance against salt or drought [4–6]. Fungal endophytes can show different behaviors depending on their environment and host plant [2, 7]. Therefore, our investigations need to consider the respective host-endophyte system. Ascomycetous fungi belonging to the *Phialocephala fortinii* s.l.–*Acephala applanata* species complex (PAC) form a major group among the dark septate endophytes (DSE) [8, 9] and predominantly colonize roots of conifers and Ericaceae throughout the Northern hemisphere [10, 11]. Different PAC genotypes were shown to belong to more than 20 cryptic species (CSP) and seven species were formally described [12]. Several PAC-host, PAC-PAC and tripartite interactions including plants and other fungi or pathogens have been investigated revealing the ecology of PAC and its competitive behavior [7, 13–19] which goes along the endophytic continuum [2]. Some PAC strains were found to be highly virulent on Norway spruce *in vitro* whereas others had no significant effect on host performance [15]. In a recent interaction experiment including two host species (*Betula pendula* and *Picea abies*) and four PAC strains, the host species as well as the PAC strain significantly influenced the results of the plant-PAC interaction [7]. However, the interaction between PAC symbionts and the host plant at the molecular level is unknown. Several RNA-Sequencing (RNA-Seq) and genome studies about pathogenic and endophytic plant-microbe interactions have already been conducted providing information about activated genes under certain conditions in fungi and oomycetes [20–24]. Various genes such as genes encoding small secreted proteins [23–25], genes involved in pathogenicity and virulence [20, 22] and in secondary metabolism [22] were found to be up-regulated in microorganisms interacting with plants. However, no study is available to our knowledge on transcriptomics of PAC interacting with one of its host plants. With this study we are going to reveal the transcriptome of the fungal symbiont during the plant-fungus interaction to better understand the mechanisms involved in this symbiosis. We feature good conditions for working with RNA-Seq on PAC as the entire and annotated genome, including functional annotations using FunCat [26] and GO [27], of the strain 6_70_1 (*Phialocephala subalpina* (CSP 6)) (70 Mbp; including 204 scaffolds) used in this study was available to us (manuscript currently in preparation). Therefore, we received very good mapping results being the basis for our further analysis. Currently, next-generation sequencing (NGS) seems to be most suitable to investigate differential gene expression due to deep sequencing coverage detecting even small differences in gene expression, simplified library construction and relatively low costs [28, 29]. Therefore, we performed an RNA-Seq study using Illumina high-throughput sequencing to reveal PAC’s transcriptome. PAC strain 6_70_1 induced by the host species *Picea abies* was investigated and fungal gene expression examined in a time course in comparison to a control without host plant. With this study we aim to answer two questions which will shed light on the function of PAC. (i) Which genes or processes are differentially expressed (DE) over time and which function do they fulfill? (ii) Do DE genes correspond to genes already found in other endophytic, pathogenic or saprotrophic fungi?

## Materials and Methods

### Experimental Setup

The experiment was setup as a time course with seven points of time (days of harvest after the day of induction: 1, 2, 3, 4, 7, 11, 18), two treatments and two biological replicates (three for day 3 to account for within replicates variability) prepared for each point of time per treatment (resulting in 15 samples per treatment and 30 in total). The treatment factor included fungal cultures which were induced with sterile Norway spruce seedlings and a control treatment without plant induction. Since plant defense and also colonization by PAC can occur within hours it was decided to start harvesting after 24 h [30].

### Culture Conditions and Harvest

100-ml Erlenmeyer-flasks containing 50 ml liquid Pachlewski media (concentration per liter: 7.3 mM KH_2_PO_4_, 5 mM (D+)-Glucose, 2.7 mM C_4_H_12_N_2_O_6_, 7.3 mM MgSO_4_ *7 H_2_O, 2.9 mM thiamine-HCL, 1 ml tap water (Zurich City) to account for a trace-element solution) were inoculated with agar plugs from actively growing mycelium of strain 6_70_1 (CSP 6, *Phialocephala subalpina*) [12], grown on 1.5%-Pachlewski agar plates. In contrast to other PAC strains, strain 6_70_1 was shown to significantly reduce biomass of *Picea abies* compared to biomass of un-inoculated control plants (S1 Fig) [7, 15].

After two weeks of incubation at room temperature and daily shaking by hands, cultures were blended to obtain many actively growing hyphal tips, and induction occurred the same day in flasks assigned to the plant treatment. Induction occurred as follows. A small styrofoam block was mounted at the transition between root and shoot of aseptically grown *Picea abies* seedlings (Birmenstorf Tannwald, Aargau, Switzerland, 400 m NN, year 1987), and three "floating" seedlings were applied to each flask (for setup example see S2 Fig). All cultures were incubated at room temperature under natural light without shaking.

24 hours after inoculation, mycelium (ca. 80–100 mg fresh weight) of the first two replicates was harvested using a sieve, and four technical replicates per biological replicate were prepared. Samples were frozen in liquid N_2_ immediately after harvesting and stored at -80°C until further processing. At the following time points samples were treated accordingly.

### Isolation of Total RNA and Sample Selection

Samples were grinded using a cooled bead mill homogenizer (Omni Bead Ruptor Homogenizer, Omni International, Kennesaw, USA) to prevent thawing. RNA was extracted using the Qiagen RNeasy Plant Mini Kit (Qiagen, Hilden, Germany) according to the manufacturer`s protocol. To completely remove genomic DNA from the samples the Qiagen RNase-Free DNase Set (Qiagen, Hilden, Germany) was used during RNA extraction. RNA quality and quantity were determined using the 2100 Bioanalyzer, Eukaryote Total RNA Nano Kit (Agilent Technologies, Waldbronn, Germany) and Qubit® 2.0 Fluorometer (Invitrogen, USA) respectively and samples for Illumina sequencing were selected accordingly.

### Preparation of cDNA Libraries and Illumina High-Throughput Sequencing

RNA was processed at the Quantitative Genomics Facility (QGF), Department of Biosystems Science and Engineering (BSSE), ETH Zurich (Basel, Switzerland) using the Illumina TruSeq RNA Sample Prep Kit v2. Samples were sequenced on two lanes (each with 15 samples) in a high output sequencing run on a HiSeq 2000 Illumina Sequencer and 50 bp single reads were generated (for sample statistics see S3 Fig). The raw de-multiplexed reads are available on the European Nucleotide Archive (ENA, www.ebi.ac.uk/ena) under the accession number PRJEB12610.

### Data Quality Assessment and Bioinformatics

Illumina sequence reads were analyzed for their quality and adjusted using the FASTX-Toolkit. The FASTX Artifacts Filter was used to eliminate reads containing artifacts such as poly-A regions. Most of the reads containing artifacts have been eliminated by Illumina itself already. The FASTQ Quality Filter set to a minimum quality score threshold of 20 and a minimum read length of 47 was used to eliminate low quality reads. The FASTX Trimmer served to eliminate single bases showing very low quality in all reads.

### Reference Genome

The genome of *Phialocephala subalpina* strain 6_70_1 [12], the same strain as the one used in this study, has previously been sequenced using 454 pyrosequencing at 25x coverage, resulting in 204 scaffolds of 69.7Mb size in total. Gene models were created using automatic prediction and subsequent manual curation. Functional annotation was done using FunCat/Pedant [26] developed at Helmholtz Zentrum Munich (manuscript currently in preparation).

### Read Mapping and Counts Table

Reads were mapped to the annotated reference genome of strain 6_70_1 (will be provided upon request). Mapping was done with the software Bowtie 2 [31]. The counts table was generated using BEDTools with a quality score threshold of 40 [32]. For bioinformatics pipeline please see S1 Table.

### Differential Gene Expression Analysis

Differential gene expression analysis was performed using the edgeR [33] which is part of the Bioconductor software implemented in R [34]. Data were analyzed for differential gene expression using the GLM approach considering biological replicates in edgeR [33]. The GLM approach considers the time course of the experiment in contrast to the classical approach where genes are compared tagwise between control and treatment per day. Count data were loaded into an edgeR library in R. Low expression tags with a cutoff value > = 3 were filtered out. Data were used to create a DGEList (Digital Gene Expression List). Library size was calculated, data normalized and a normalized counts table was generated. Differential gene expression was calculated between two groups (control versus treatment) taking into account the experiment was setup as a time course. However, the logFC was calculated for each day separately between control and treatment but only one *p*-value resulted for each gene. *P*-values for significantly DE genes over time were set to ≤ 0.01 and false discovery rate (FDR) to ≤ 0.05. The Benjamini-Hochberg method was used to adjust for multiple hypothesis testing of the resulting *P*-values [35].

### Gene Set Enrichment Analysis

To investigate enriched occurrence of certain biological processes in DE genes we performed a Gene Ontology (GO) terms [27] enrichment analysis using the package GOstats in R [36]. Genes were regarded as enriched if the *P*-value was below the threshold of 0.05.

## Data Validation Using Real-Time RT-PCR

**Fig 1:**
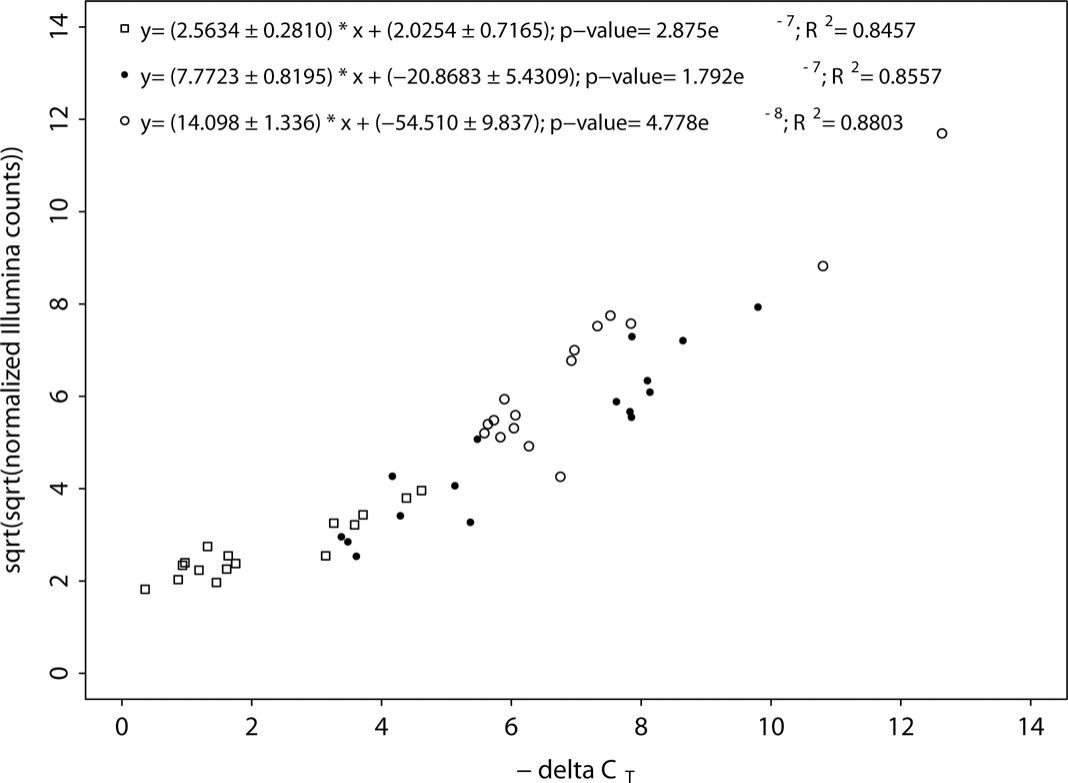
Validation of Illumina data using real-time RT PCR. Sqrt-transformed, normalized Illumina counts were plotted against negative ∆C_T_ values for correlation. Colors represent different target genes (open rectangle = PAC_1440, closed circle = PAC_18739 and open circle = PAC_7783) which were validated. Housekeeping gene PAC_19651 served as reference gene.

Four genes (PAC_01440, primer f: TCTCCTCCAACGACCTGAGT, r: AATTTGTGTGGGTTGATGGCTG; PAC_07783, primer f: GCGGAGTTGTATGGGAGCTT, r: CCACACAGCAATGAACGCAA; PAC_14815, primer f: AAACACACCAAACGCTACCAATACG, r: CCGCCTCCGCAATGTCACTA and PAC_18739, primer f: TTCGACTATCACAGGACGCC, r: AAGATTTCTGCACCGACAAGC) which are significantly DE over time compared to the control were chosen for data validation using real-time RT-PCR and four housekeeping genes (PAC_00931, primer f: AAGCCATGCGAGGAGGATATG, r: GAGAGACGACCCTTGCTTGT; PAC_04323, primer f: CGTGCTGAAGAGGTCCAAGA, r: AGAGGATCGGAGGCTCTCAG; PAC_05494, primer f: ATTCCTGGCGAACAACCCAT, r: ATCCGTGAAGCCGTTGATGA; PAC_19651, primer f: ACCCACTCGCTCAGAACTTG, r: ATGCCACACGAGGTCTTGAG), whereas PAC_19651 was eventually used as reference gene. The logFC between control and treatment for the selected genes was approximately between two and four. Primers were designed at equally high expressed regions within the coding sequence of the gene and at the highest expressed location. RNA (500 ng per sample) from two biological replicates was used for cDNA synthesis using the Quantitect Reverse Transcription Kit (Qiagen, Hilden, Germany) according to the manufacturer’s protocol. Real-time PCR was performed using the KAPA SYBR FAST qPCR kit (Kapa Biosystems, Inc., Wilmington, USA) with 1:3 diluted cDNA including no-RT and H_2_O controls. All steps were performed following the manufacturer’s protocol and run on a 7500 Fast Real-Time PCR System (Life Technologies, Grand Island, USA). Expression levels were calculated using—∆C_T_ values and normalized Illumina counts (Fig 1). Mean amplification efficiency of real-time RT PCR was 95%.

## Results

### Data Quality and Mapping

**Fig 2:**
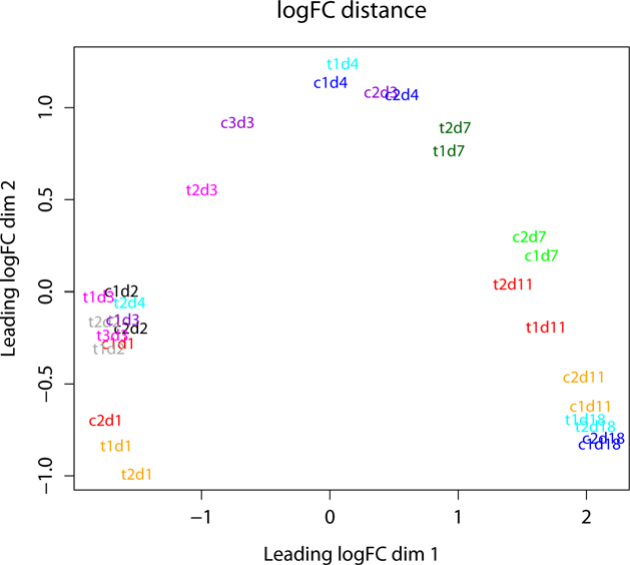
MDS plot showing sample relations. Sample relations are plotted using a multidimensional scaling plot (MDS) generated with edgeR showing the variability between replicates and different treatments in log2-fold-change distance. The axes represent gene expression levels between the different experimental factors. Samples from days 1, 7, 11 and 18 show best separation between treatments and best accordance of replicates. “c” = control, “t” = treatment and d1 –d18 = days after induction with the host plant.

Library sizes ranged from 10,000 000 to 20,000 000 reads (raw data) and samples run in different lanes during Illumina sequencing did not show any bias depending on the lane. The cleaned (filtered and trimmed) reads had a mean phred quality score of 42. Using bowtie 2 [31] 90% of the quality filtered reads could be mapped on as single position on the reference genome and transcriptome with a phred quality score of 42. Reads which mapped at more than one position in the genome or with lower quality were discarded. Variation between libraries (between treatments and biological replicates) is shown in Fig 2. For days 3 and 4 replicates were very heterogeneous and variability within treatments was higher than between treatments. Days 1, 7, 11 and 18 showed a clear differentiation between treatments and much lower variability between replicates (Fig 2).

### Gene Expression over Time

**Fig 3:**
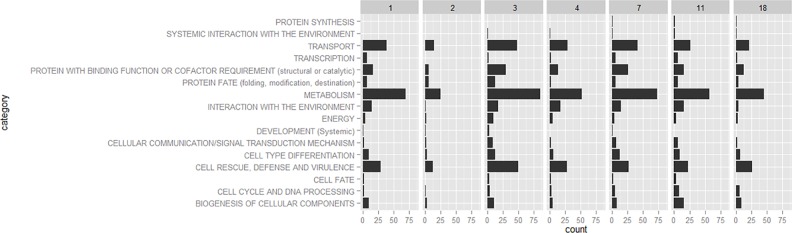
Differentially expressed genes. Number of DE genes belonging to the main functional categories according to FunCat annotations over time. Most genes fall into more than one functional category and therefore, contribute to several categories. LogFC cutoff was between -2 and 2 therefore DE genes within this range are displayed only.

Analyzing the data using the edgeR GLM approach [33] from the Bioconductor software in R [34] resulted in 2549 DE genes between control and treatment over time. This represents a percentage of 16.25% of all the genes expressed in this study (15690 genes). Of the DE genes 1124 out of 2549 carried functional annotations according to the FunCat [26] scheme (see S2 Table). The main functional categories which showed most activity are ‘metabolism’ (844 DE genes), ‘transport’ (375 DE genes) and ‘cell rescue, defense and virulence’ (332 DE genes). Those main functional categories stayed most active until day 18. Whereas day 3 showed most DE genes between control and treatment day 2 showed the least DE genes (Fig 3, Tables 1 and 2). A complete shift of main functional categories between days could not be observed but rather the same pattern for each day differing in intensity (Fig 3).

**Table 1:** FunCat functional main categories in DE genes and their number summarized over all days.

FunCat Functional Main Categories	# of Genes
BIOGENESIS OF CELLULAR COMPONENTS	152
CELL CYCLE AND DNA PROCESSING	146
CELL FATE	67
CELL RESCUE, DEFENSE AND VIRULENCE	332
CELL TYPE DIFFERENTIATION	132
CELLULAR COMMUNICATION/SIGNAL TRANSDUCTION MECHANISM	75
DEVELOPMENT (Systemic)	17
ENERGY	110
INTERACTION WITH THE ENVIRONMENT	156
METABOLISM	844
PROTEIN FATE (folding, modification, destination)	145
PROTEIN SYNTHESIS	16
PROTEIN WITH BINDING FUNCTION OR COFACTOR REQUIREMENT (structural or catalytic)	398
SYSTEMIC INTERACTION WITH THE ENVIRONMENT	5
TRANSCRIPTION	114
TRANSPORT	375
TRANSPOSABLE ELEMENTS, VIRAL AND PLASMID PROTEINS	1
no category	1425

**Table 2:** Top 20 genes most significantly differentially expressed between control and treatment over time according to the edgeR GLM method. LogFC = log Fold Change between control and treatment, FDR = False Discovery Rate, CPM = Counts Per Million.

Gene Code	Functional Gene Annotation	Funcat Main Category	Description	logFC Day 1	logFC Day 2	logFC Day 3	logFC Day 4	logFC Day 7	logFC Day 11	logFC Day 18	Highest logFC at day	P-Value	FDR	logCPM
PAC_01440	uncharacterized protein	no category	na	-1.530341617	-0.58282662	-1.520190979	-1.254780778	-1.952812207	-2.601939676	-2.379149015	11	1.26E-28	1.97E-24	2.28205968
PAC_18737	uncharacterized protein	no category	na	-1.460377737	-1.932448084	-3.042814052	-1.689044419	-5.929322825	-3.5344891	-2.08347107	7	5.20E-27	4.06E-23	6.08098427
PAC_18740	related to RTG2—retrograde regulation protein	Metabolism	regulation of C-compound and carbohydrate metabolism	0.698306005	-0.720126699	-0.461580243	0.570595339	-5.077109152	-3.82182404	-1.581386444	7	1.54E-22	8.03E-19	2.60302769
		Transcription	transcriptional control											
		Cellular communication/Signal transduction mechanism	cellular signalling											
PAC_18741	uncharacterized protein	no category	na	-2.353046393	-0.897116249	-0.346015729	0.361017913	-3.997893646	-3.658711825	-1.96572373	7	2.86E-21	1.12E-17	7.42502253
PAC_14815	related to carboxylic acid transporter protein	Metabolism	C-compound and carbohydrate metabolism	0.635168394	0.573584713	0.508541284	1.073229882	3.377508291	4.401268268	1.266184716	11	2.06E-20	6.43E-17	7.70917001
		Transport	C-compound and carbohydrate transport											
PAC_12637	uncharacterized protein	no category	na	4.075938007	1.058656198	5.02499936	8.126108748	2.881261253	5.629728592	1.56510553	4	2.19E-19	5.69E-16	4.20276895
PAC_18646	related to RTG2—retrograde regulation protein	Metabolism	regulation of C-compound and carbohydrate metabolism	-1.158896633	-0.331223689	-0.540235821	-0.578489183	-2.808185905	-1.992957635	-1.344801346	7	6.27E-19	1.40E-15	5.28752859
		Transcription	transcriptional control											
		Cellular communication/Signal transduction mechanism	cellular signalling											
PAC_18722	related to pyridine nucleotide-disulphide oxidoreductase AMID-like	no category	na	5.008129888	0.978113693	4.210387116	6.839054342	1.863088733	1.811797737	0.589328499	4	9.03E-19	1.76E-15	6.0509731
PAC_05253	related to multifunctional beta-oxidation protein	Metabolism	fatty acid metabolism	1.929781654	0.59085492	3.113251607	5.007505449	2.471273337	2.094124569	0.290314498	4	2.05E-18	3.56E-15	3.22174867
		Energy	oxidation of fatty acids											
		Biogenesis of cellular components	cell wall											
PAC_05250	uncharacterized protein	Metabolism	amino acid metabolism	2.919419975	0.421120149	3.783338365	5.780790297	2.537297889	2.139438176	0.602759102	4	1.96E-17	2.94E-14	5.77465961
PAC_18739	related to potassium channel	Transport	cation transport (H+, Na+, K+, Ca2+, NH4+, etc.)	-1.267387809	-0.985549625	-0.990733172	-1.127873871	-3.511690595	-2.380019083	-1.051713858	7	2.07E-17	2.94E-14	5.93554796
		Interaction with the environment	homeostasis of metal ions (Na, K, Ca etc.)											
PAC_05564	uncharacterized protein	no category	na	-0.400770869	1.148804727	0.647993787	1.267004012	3.32926504	2.491822987	1.740593727	7	2.43E-17	3.16E-14	4.42158449
PAC_17336	related to heat shock protein Hsp30-like	Cell rescue, defense and virulence	heat shock response	-1.671776792	1.443486204	1.143889065	1.91039193	3.776576519	6.246180545	0.985783377	11	6.85E-17	8.22E-14	10.1520462
PAC_18736	uncharacterized protein	no category	na	0.068972702	-2.026494886	-3.262050919	-1.946441446	-5.548463251	-3.65342491	-2.047412827	7	3.61E-16	4.03E-13	5.25265106
PAC_07783	related to major facilitator MirA	Metabolism	secondary metabolism	0.130463243	0.481599492	1.962441702	3.793612792	1.877518856	2.138736193	0.579514161	4	1.04E-15	1.08E-12	8.06436576
		Transport	cellular import											
		Cell rescue, defense and virulence	detoxification											
		Interaction with the environment	homeostasis of metal ions (Na, K, Ca etc.)											
PAC_09616	probable protein involved in intramitochondrial protein sorting	Protein synthesis	aminoacyl-tRNA-synthetases	0.082777886	-0.119571057	0.294293519	1.164513731	3.262961174	3.582360128	2.507893816	11	1.35E-15	1.27E-12	6.60765035
		Protein fate (folding, modification, destination)	protein targeting, sorting and translocation											
PAC_05251	related to long-chain-fatty-acid-CoA ligase	Metabolism	lipid, fatty acid and isoprenoid metabolism	2.390121052	0.756668416	3.282507269	5.921210147	1.642555049	0.714536337	0.622661201	4	1.38E-15	1.27E-12	5.3099434
		Protein with binding function or cofactor requirement (structural or catalytic)	nucleotide/nucleoside/nucleobase binding											
PAC_05249	related to multidrug resistance protein	Metabolism	phosphate metabolism	1.524016758	0.22724417	2.58496999	4.502200045	1.051518046	0.964808963	0.125093106	4	8.43E-15	7.31E-12	5.43237949
		Transport	ABC transporters											
		Cell rescue, defense and virulence	detoxification by export											
		Protein with binding function or cofactor requirement (structural or catalytic)	ATP binding											
PAC_18645	related to lysophospholipase	Metabolism	lipid, fatty acid and isoprenoid metabolism	-4.58643436	-1.077253484	-1.393095159	-0.461916451	-4.524263932	-2.582037231	-1.302095297	1	2.44E-14	2.00E-11	4.52940038
		Cell cycle and DNA processing	meiosis											
		Transcription	transcriptional control											
		Cell type differentiation	development of asco- basidio- or zygospore											
PAC_13159	related to multidrug resistance protein	Metabolism	phosphate metabolism	1.135192768	-0.039228864	2.231865693	4.455920424	1.209089413	1.5699544	0.084038762	4	3.39E-14	2.65E-11	6.63354686
		Protein with binding function or cofactor requirement (structural or catalytic)	ATP binding											
		Transport	ABC transporters											
		Cell rescue, defense and virulence	detoxification by export											
		Interaction with the environment	chemoperception and response											

### Most Significantly DE Genes—Top 20

**Fig 4:**
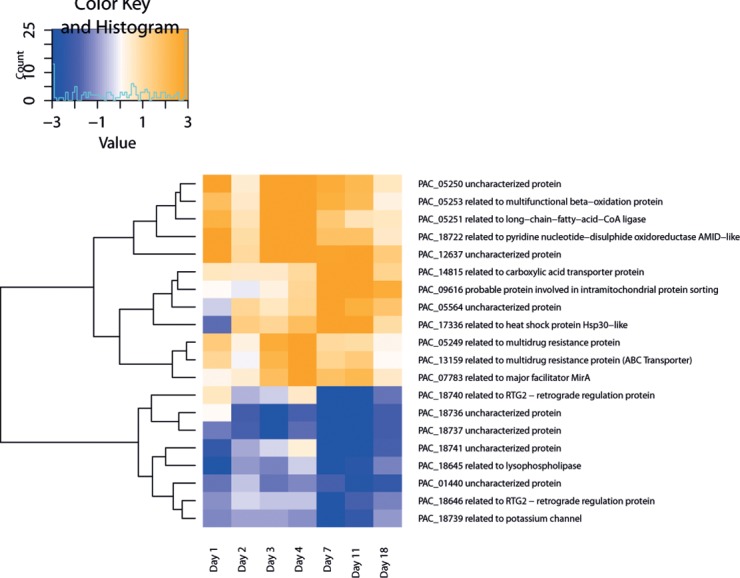
Top 20 differentially expressed genes. Heat map depicting the Top 20 genes including their main functional categories. Gene expression in blue shows down-regulated and in orange up-regulated genes. Genes were clustered by their expression pattern. The transcription level is depicted in logFC values. Genes displayed in blue are downregulated, therefore expressed in favor of the control and genes depicted in orange are expressed in favor of the treatment. Count corresponds to the number of reads covering the gene model at each timepoint.

In Table 2 the top 20 DE genes which show highest log-fold change (logFC) between control and treatment over time are listed with functional gene annotations assigned in FunCat [26]. Values showing a positive logFC value symbolize higher expression of the treatment compared to the control and vice versa. The most frequent functional categories within these Top 20 genes were ‘metabolism’, ‘transport’ and ‘cell rescue, defense and virulence’ (Table 2). Day 4 is the time point where most DE genes have their highest logFC, i.e. 96 hours after initiating the plant-fungus interaction (Table 2). Eleven genes of these most significantly DE genes belong to two gene clusters evident by the ‘Gene Code’ in Table 2. Five of these belong to one cluster (PAC_18736—PAC_18741, except for PAC_18738, DE only) and four to another one (PAC_05249—PAC_05253 except for PAC_05252, DE only). There are seven genes which could not be assigned to any category. Two main clusters in terms of gene regulation could be found and therefore the heatmap appears to be divided into two parts (Fig 4). Twelve genes happened to be mostly up-regulated upon induction of the host plant (including all genes of “cluster 2”) whereas eight genes were down-regulated (including another cluster of two genes, PAC_18645/6) (Fig 4). Main functional categories which only appear in the up-regulated cluster were: ‘cell rescue, defense and virulence’, ‘protein with binding function or cofactor requirement’, ‘protein fate’, ‘protein synthesis’, ‘biogenesis of cellular components’ and ‘energy’. In contrast functional main categories under the Top 20 presented exclusively by down-regulated genes were: ‘transcription’, ‘cellular communication/signal transduction mechanism’, ‘cell cycle and DNA processing’ and ‘cell type differentiation’ (Fig 4).

### Genes Indicating PACs Lifestyle

Within the main functional category of ‘metabolism’ (844 DE genes) the two functional sub-categories ‘C-compound and carbohydrate metabolism’ (228 DE genes) and ‘secondary metabolism’ (159 DE genes) were mainly activated. This group contains many genes predicted to be involved in cell wall degradation, for instance pectinesterase, pectate lyase, feruloyl esterase, cellulase, β-glucosidase, mannan endo-1,4-β-mannosidase and cellobiose dehydrogenase to mention a few examples [37–40]. DE genes were found to be involved in tyrosin degradation and therefore being responsible for melanin production, a substance which is typical for DSE [41].

The main functional group of ‘transport’ (375 DE genes) included the two sub-categories ‘C-compound and carbohydrate transport’ (88 DE genes) and ‘electron transport’ (78 DE genes) which were represented with most DE genes in this functional group. In this category we found significant activity in the transcriptome for major facilitator-superfamily (MFS) and ATP-binding cassette (ABC) transporters which are very often involved in cellular detoxification processes [39, 42, 43]. Also genes predicted to be involved in hexose transport were found within the DE genes.

For ‘cell rescue, defense and virulence’ (332 DE genes) we could find three main functional sub-categories with highest numbers of DE genes. Those are ‘detoxification’ (62 DE genes), ‘stress response’ (33 DE genes) and ‘disease, virulence and defense’ (31 DE genes). Within those groups many genes predicted to be involved in detoxification or degradation processes could be detected. We found ATP-binding cassette (ABC) transporters, which were amongst others represented by the aflatoxin efflux pump AFLT, which are mainly involved in detoxification [39, 43]. Glutathione S-transferase (GST) of which two related genes could be detected in PACs DE transcriptome functions in the detoxification of xenobiotics and peroxides [44, 45]. Additionally, genes predicted to be involved in cytochrome P450 fell into this main category as well.

### Gene Set Enrichment Analysis

According to our gene set enrichment analysis using GOstats 178 GO categories of the class “biological process” appeared to be enriched within our 2549 DE genes summarized over all days (see S3 Table). Looking at the ten genes with the lowest *p*-value per day the following biological processes could be identified to be enriched (see S4 Table). At day 1 especially iron- and carbohydrate transport related biological processes were enriched. Most significant biological processes at day 2 were activities involved in vitamin B_6_ and antibiotics metabolism. After 3 days of induction with the host plant the fungal transcriptome showed enriched activity mainly in the areas of iron metabolic and transport processes. At day 4 biological processes exclusively involved in iron metabolism and metal ion homeostasis were enriched. Day 7 is dominated by processes being involved in cell aging and iron metabolism whereas day 11 is showing solely activities in iron transport and metabolic processes and ion homeostasis. At day 18 after induction with the host plant the fungus actively tried to evade the immune response of the host and other processes as amino acid catabolism were significantly enriched. Cellular response to iron starvation seemed to be the major issue for the fungus within the time frame of the experiment.

## Discussion

DE genes were found predominantly in the main functional categories ‘metabolism’, ‘transport’ and ‘cell rescue, defense and virulence’. There was no general shift in those main categories observed over the time span of the experiment and genes in those categories stayed differentially expressed until day 18. Considerable variation among replicates especially at day 3 and 4 could be a possible explanation why the main functional groups appeared to be expressed equally well over the time span of the experiment. Another reason why only little change could be observed over time might be the fact that the infection process went on during the entire experiment (Fig 3). This would be in concordance with our observation that infection of the host plant with PAC strain 6_70_1 in the liquid culture setup takes place from day 1 on with linearly increasing colonization density of the host plants’ roots until the end of the experiment (Reininger V., personal observation). Fungal hydrolases which are involved in metabolic functions were found to be highly up-regulated in another study investigating a pathogenic mutant form ∆*sakA* of *Epichloë festucae* [46]. From the biological point of view it seems to be reasonable to find metabolic functions most active as the fungus starts changing its live form from a saprotrophic (in the media) to an at least partially biotrophic form in the host plant. The fact that many cell wall degrading enzymes are activated can be explained by the colonization process of the fungus. Additionally, PAC seems to go through a glucose depletion stage during host colonization indicated by up-regulation of genes related to carbohydrate transport and metabolism and the induction of cell wall degrading enzymes (CWDE). The same pattern of up-regulated genes could be detected in *Pirifomospora indica* on dead root tissue 5 days after induction [24]. The high number of genes predicted to be involved in cell wall degradation and among those genes involved in metabolic functions, could be a sign of a pathogenic life form of PAC strain 6_70_1 in symbiosis with *P*. *abies* [39, 40]. In contrast Zuccaro *et al*. [24] found more CWDE to be active in saprotrophic fungi compared to pathogenic ones.

Furthermore, the plant very likely switched on its defense system and PAC might have been confronted as well with phenolic compounds or terpenoids produced by the host plant as PAC shows high activity in genes related to detoxification and degradation processes of foreign substrates thereby involving many transporters amongst others being responsible for export of plant related toxins. Shown by the up-regulation of ABC and major facilitator transporters the fungus protects itself against chemical compounds e.g. belonging to the terpenoids or the aromatic phenolic compounds and the plant defense system [42, 47, 48]. Many major facilitator transporters are involved in detoxification, exporting defense molecules such as terpenoids or aromatic phenolic compounds. Phenolic compounds can be secreted by induction if the pathogen starts attacking the host plant or on the other hand they can be available as performed phenolics which means they are produced by the plant independently from the colonization of the pathogen. In this case the chemicals concentration won’t be high enough to be harmful to the pathogen [48]. Also in the study of Eaton et al. [46] fungal transporters in the pathogenic ∆*sakA* mutant were drastically up-regulated. The up-regulation of transporters in PAC could be a hint that PAC is rather escaping the plant defense system than actively defending itself against the host plant by producing mycotoxins. However, metabolic processes of macrolactones were found to be enriched at day 2. The macrocyclic lactone epothilone is known to have a phytotoxic effect on tomatoes [49] and from the transcriptome data of this study we assume it was biosynthesized by PAC two days after induction with *Picea abies*.

Looking at enriched DE genes gives more insight into the most important processes during the PAC—host (*Picea abies*) interaction. According to the gene set enrichment analysis using GO categories iron seems to be a major player in this induction process (see S4 Table). Especially the top 10 (given by lowest p-values) at days 3, 4 and 11 exclusively show biological processes related to iron. During host colonization PAC is confronted with an iron depletion as the host will assimilate iron as well and therefore the fungus actively needs to acquire iron from its environment/host especially from day 3 on up to day 11 (see S4 Table). Iron mostly exists in a complexed form and free iron is only available in very limited amounts [50]. Therefore, during infection host and pathogen are competing for iron and fungal pathogens have evolved different strategies to capture iron from the host [50–52]. This result could point towards a pathogenic lifestyle as for a successful fungal pathogen iron is a key compound to be assimilated from the host by different mechanisms such as iron uptake from ferritin or via siderophores [52–54]. On the other hand siderophores are also produced e.g. by fungal endophytes or mycorrhiza in interaction with their hosts and therefore also play a role in neutral or mutualistic symbiotic interactions [55, 56]. However, siderophore-iron transport was one of the categories found to be differentially expressed in PAC. Strain-dependent production of several types of siderophores by PAC was already confirmed before [57]. Bacterial siderophores in host plants were also shown to have the ability to activate a plant immune response [58, 59]. This observation matches with the expression pattern in strain 6_70_1 as the ‘active evasion of host immune response’ follows several processes related to iron (see S4 Table).

PAC is a melanin producing dark septate endophyte and genes related to tyrosine metabolism were found to be up-regulated upon induction by the host, thereby additionally increasing the metabolic activity of strain 6_70_1. According to the literature melanin is known to be involved in pathogenicity [51, 60].

We need to consider that we have not extracted the fungus from the roots but the two symbionts were in contact over the liquid media only. This is an important point to be aware of when interpreting the results. The gene expression pattern of PAC might change completely already having first contact with its hosts roots. However, it could also be the case that PAC will change its expression again when colonizing the roots eventually.

In conclusion we could demonstrate that PAC is very likely on the edge between being a harmless endophyte and a pathogen. Strain 6_70_1 used in this experiment was tested to behave pathogenic on *Picea abies* in a former experiment investigating plant biomass after induction with PAC compared to a mock-inoculated control (S1 Fig). We found indicators also in this transcriptome study pointing towards a pathogenic lifestyle of this strain [7, 15]. For instance the up-regulated tyrosine metabolism or the putative production of antibiotics at day 2. On the other hand according to Zuccaro *et al*. [24] the strong activity of CWDE rather confirms that strain 6_70_1 behaves endophytic on *Picea abies*. The very distinct iron metabolism could be a sign for either a pathogenic or an endophytic interaction between these two symbionts.

This study is the first one investigating PACs transcriptome and gives us an insight into its ambivalent lifestyle between being a pathogen and a benign endophyte.

## Supporting Information

S1 Fig Plant biomass in [g] according to inoculation with PAC strain 6_70_1 in comparison to control plants without fungal inoculation. According to an ANOVA analysis the difference in plant biomass is highly significant (*p*-value = 3.64e-05) between the two treatments.
(PDF)

S2 Fig Schematic illustration of the inoculation system pictured here with only two Styrofoam blocks per flask in comparison to three in the experiment. (JPG)

S3 Fig Statistics of the Illumina sequencing run including all samples. (PDF)

S1 Table Bioinformatics pipeline in shellscript format. (DOCX)

S2 Table Differentially expressed genes including FunCat annotations. (XLSX)

S3 Table Enriched genes according to our gene set enrichment analysis using GOstats. (XLSX)

S4 Table Ten genes each with the lowest *p*-value per day according to our enrichment analysis. (XLSX)
