# Supplementary figures and images for: Analysis of the Phialocephala subalpina Transcriptome during Colonization of Its Host Plant Picea abies
Source: PLoS One. 2016 Mar 8;11(3):e0150591. doi: 10.1371/journal.pone.0150591 (PMC4783019; doi:10.1371/journal.pone.0150591)

# ***Picea abies* Biomass**

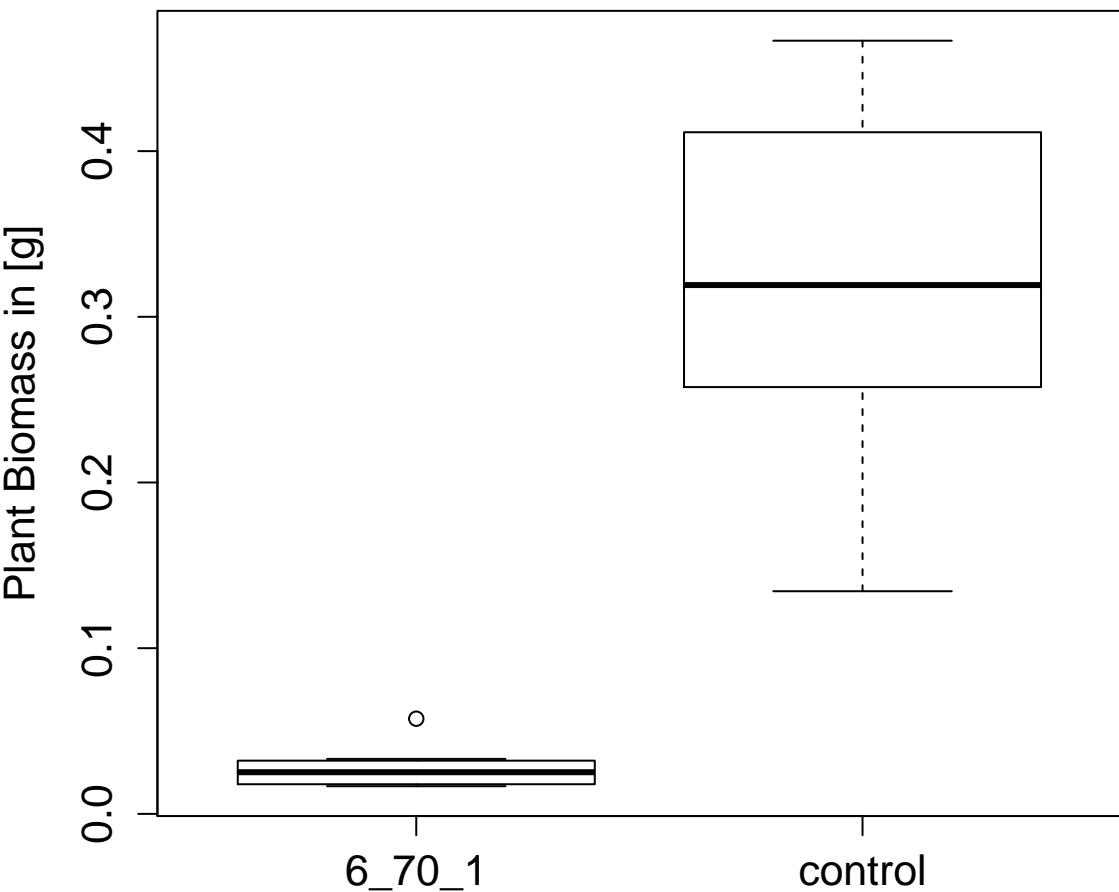

Supplement: S1 Fig — According to an ANOVA analysis the difference in plant biomass is highly significant (p-value = 3.64e-05) between the two treatments. (PDF) [file pone.0150591.s001.pdf]

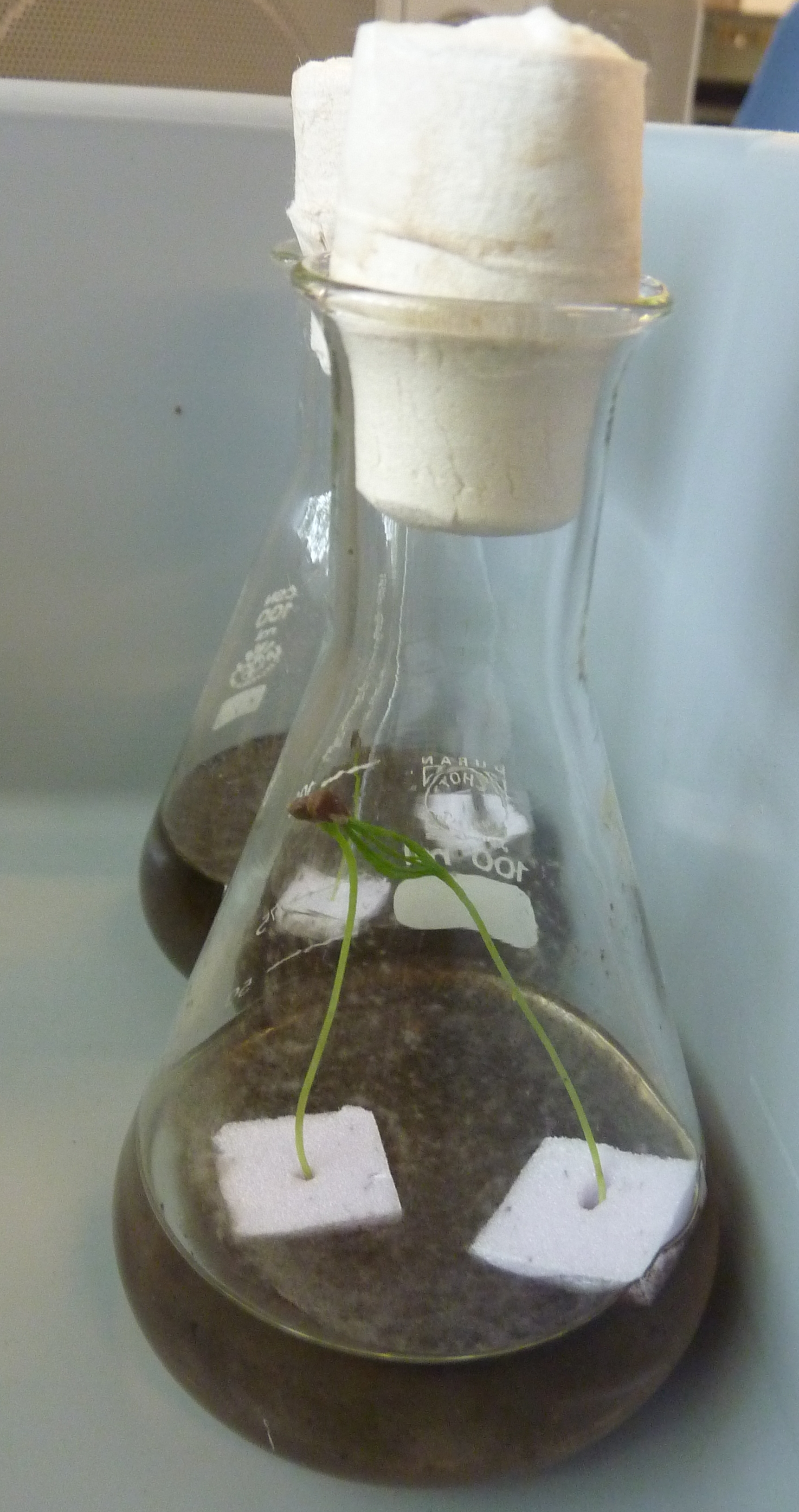

Supplement: S2 Fig — (JPG) [file pone.0150591.s002.jpg]
