# Supplementary material for: Analysis of the Phialocephala subalpina Transcriptome during Colonization of Its Host Plant Picea abies
Source: PLoS One. 2016 Mar 8;11(3):e0150591. doi: 10.1371/journal.pone.0150591 (PMC4783019; doi:10.1371/journal.pone.0150591)

Flowcell: D1FBTACXX

Barcode lane statistics

Lane	Sample ID	Index	Description	Control	Yield (Mbases)	# Reads (PF)	% PF	# Reads	% of raw clusters per lane	% of >= Q30 Bases (PF)	Mean Quality Score (PF)
5	BSSE_QGF_9345_121130_SN792_0189_AD1FBTACXX_5	NoIndex	PhiX	Y	6,499	129,990,628	94.88	137,005,299	100	95.82	37.79
7	BSSE_QGF_9079_121130_SN792_0189_AD1FBTACXX_7	CGATGT	1_C_d1_1_1	N	413	8,266,455	85.57	9,660,459	3.82	91.62	36.17
7	BSSE_QGF_9080_121130_SN792_0189_AD1FBTACXX_7	TGACCA	4_C_d1_2_2	N	810	16,208,417	84.26	19,236,194	7.61	91.51	36.14
7	BSSE_QGF_9081_121130_SN792_0189_AD1FBTACXX_7	ACAGTG	10_T_d1_2_2	N	477	9,548,964	84.35	11,320,645	4.48	91.51	36.14
7	BSSE_QGF_9082_121130_SN792_0189_AD1FBTACXX_7	GCCAAT	11_T_d1_3_1	N	478	9,558,107	83.71	11,418,118	4.52	91.41	36.11
7	BSSE_QGF_9083_121130_SN792_0189_AD1FBTACXX_7	CAGATC	14_C_d2_1_2	N	375	7,509,783	84.7	8,866,332	3.51	91.55	36.15
7	BSSE_QGF_9084_121130_SN792_0189_AD1FBTACXX_7	CTTGTA	18_C_d2_3_2	N	460	9,207,540	83.87	10,978,347	4.34	91.47	36.13
7	BSSE_QGF_9085_121130_SN792_0189_AD1FBTACXX_7	AGTCAA	19_T_d2_1_1	N	680	13,608,711	84.77	16,053,688	6.35	91.65	36.18
7	BSSE_QGF_9086_121130_SN792_0189_AD1FBTACXX_7	AGTTCC	24_T_d2_3_2	N	701	14,020,807	84.7	16,553,491	6.55	91.6	36.16
7	BSSE_QGF_9087_121130_SN792_0189_AD1FBTACXX_7	ATGTCA	26_C_d3_1_2	N	696	13,924,003	84.48	16,482,011	6.52	91.61	36.17
7	BSSE_QGF_9088_121130_SN792_0189_AD1FBTACXX_7	CCGTCC	27_C_d3_2_1	N	663	13,270,566	84.1	15,779,508	6.24	91.33	36.07
7	BSSE_QGF_9089_121130_SN792_0189_AD1FBTACXX_7	GTCCGC	30_C_d3_3_2	N	585	11,692,979	85.04	13,749,975	5.44	91.51	36.13
7	BSSE_QGF_9090_121130_SN792_0189_AD1FBTACXX_7	GTGAAA	32_T_d3_1_2	N	589	11,788,769	85.06	13,859,357	5.48	91.63	36.17
7	BSSE_QGF_9091_121130_SN792_0189_AD1FBTACXX_7	ATCACG	34_T_d3_2_2	N	723	14,461,657	83.76	17,265,588	6.83	91.45	36.12
7	BSSE_QGF_9092_121130_SN792_0189_AD1FBTACXX_7	TTAGGC	36_T_d3_3_2	N	709	14,176,041	84.41	16,794,267	6.65	91.29	36.06
7	BSSE_QGF_9093_121130_SN792_0189_AD1FBTACXX_7	ACTTGA	38_C_d4_1_2	N	843	16,865,310	83.13	20,287,874	8.03	91.46	36.12
7	lane7		Undetermined	N	633	12,657,283	36.78	34,413,494	13.62	83.72	33.85
8	BSSE_QGF_9094_121130_SN792_0189_AD1FBTACXX_8	GATCAG	42_C_d4_3_2	N	1,012	20,237,288	86.99	23,263,925	9.46	92.77	36.52
8	BSSE_QGF_9095_121130_SN792_0189_AD1FBTACXX_8	TAGCTT	45_T_d4_2_1	N	716	14,329,863	86.78	16,512,863	6.71	92.76	36.52
8	BSSE_QGF_9096_121130_SN792_0189_AD1FBTACXX_8	GGCTAC	48_T_d4_3_2	N	802	16,034,174	87.89	18,243,457	7.42	92.84	36.53
8	BSSE_QGF_9097_121130_SN792_0189_AD1FBTACXX_8	GTGGCC	52_C_d7_2_2	N	702	14,047,267	88.09	15,946,495	6.48	92.82	36.53
8	BSSE_QGF_9098_121130_SN792_0189_AD1FBTACXX_8	GTTTCG	54_C_d7_3_2	N	632	12,636,962	87.39	14,460,421	5.88	92.87	36.55
8	BSSE_QGF_9099_121130_SN792_0189_AD1FBTACXX_8	CGTACG	56_T_d7_1_2	N	647	12,939,333	87.88	14,723,866	5.99	92.88	36.55
8	BSSE_QGF_9100_121130_SN792_0189_AD1FBTACXX_8	GAGTGG	60_T_d7_3_2	N	680	13,591,164	88.95	15,279,555	6.21	93.07	36.6
8	BSSE_QGF_9101_121130_SN792_0189_AD1FBTACXX_8	ACTGAT	74_C_d11_1_2	N	780	15,593,710	87.64	17,792,914	7.23	92.94	36.58
8	BSSE_QGF_9102_121130_SN792_0189_AD1FBTACXX_8	ATTCCT	78_C_d11_3_2	N	721	14,415,267	87.32	16,508,551	6.71	92.92	36.57
8	BSSE_QGF_9103_121130_SN792_0189_AD1FBTACXX_8	CGATGT	82_T_d11_2_2	N	560	11,193,591	88.53	12,643,839	5.14	92.93	36.56
8	BSSE_QGF_9104_121130_SN792_0189_AD1FBTACXX_8	TGACCA	84_T_d11_3_2	N	592	11,837,625	87.8	13,482,489	5.48	92.87	36.55
8	BSSE_QGF_9105_121130_SN792_0189_AD1FBTACXX_8	ACAGTG	97_C_d18_1_1	N	514	10,286,111	88.1	11,675,495	4.75	92.9	36.56
8	BSSE_QGF_9106_121130_SN792_0189_AD1FBTACXX_8	GCCAAT	102_C_d18_3_2	N	356	7,113,410	87.21	8,156,645	3.32	92.72	36.5
8	BSSE_QGF_9107_121130_SN792_0189_AD1FBTACXX_8	CAGATC	106_T_d18_2_2	N	653	13,052,852	87.43	14,929,489	6.07	92.8	36.53
8	BSSE_QGF_9108_121130_SN792_0189_AD1FBTACXX_8	CTTGTA	108_T_d18_3_2	N	488	9,754,760	87.78	11,112,736	4.52	92.9	36.56
8	lane8		Undetermined	N	419	8,383,402	39.54	21,202,332	8.62	86.19	34.53

Lanes 7/8 - Summary overview

# reads, PF (lane 7, total)	196,765,390	"total" = samples + unmatched index reads
# reads, PF (lane 7, samples)	184,108,107	
# reads, PF (lane 7, samples/total)	94%	this high number reflects an optimal de-multiplexing process
# reads, PF (lane 8, total)	205,446,780	
# reads, PF (lane 8, samples)	197,063,378	
# reads, PF (lane 8, samples/total)	96%	this high number reflects an optimal de-multiplexing process

Overall number of reads passing filter is very high and average Qscore of all libraries is >36.

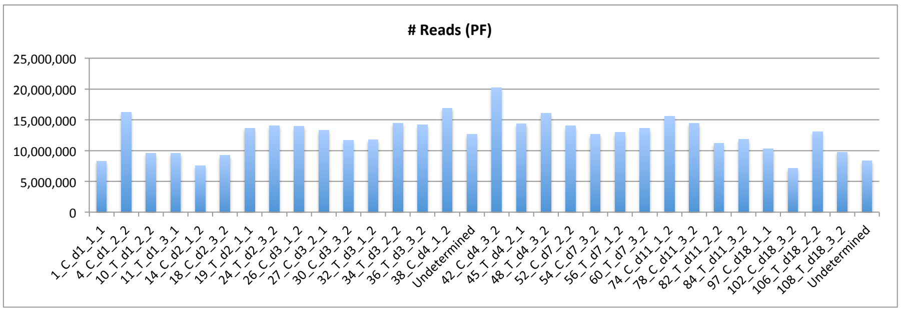

Supplement: S3 Fig — (PDF) [file pone.0150591.s003.pdf]
