# Supplementary material for: Analysis of the Phialocephala subalpina Transcriptome during Colonization of Its Host Plant Picea abies
Source: PLoS One. 2016 Mar 8;11(3):e0150591. doi: 10.1371/journal.pone.0150591 (PMC4783019; doi:10.1371/journal.pone.0150591)
Supplement: S1 Table — (DOCX) [file pone.0150591.s004.docx]

**S1 Table. Bioinformatics pipeline in shellscript format**

1. **Clean reads**

#! /bin/sh

for SMPL in `cat sample.list`

do

FILEALL=`ls data/raw_data/unzipped_raw_data/$SMPL`

FILENAME=${FILEALL##*raw_data/}

FILECORE=${FILENAME%%.fastq}

echo "=================================="

echo "start: ${FILENAME}"

fastx_artifacts_filter -Q 33 -v -i data/raw_data/unzipped_raw_data/${FILENAME} -o data/trim/${FILECORE}_art.fastq

echo "art filter done"

echo "----------------------------------"

fastq_quality_trimmer -Q 33 -t 20 -l 47 -v -i data/trim/${FILECORE}_art.fastq -o data/trim/${FILECORE}_art_q20t47.fastq

echo "q filter done"

echo "----------------------------------"

fastx_trimmer -Q 33 -v -f 4 -i data/trim/${FILECORE}_art_q20t47.fastq -o data/trim/${FILECORE}_art_q20t47_trimf4.fastq

echo "trim finished"

echo "=================================="

done

1. **Run mapping**

#! /bin/sh

echo ""

for SMPL in 9079 9080 9081 9082 9083 9084 9085 9086 9087 9088 9089 9090 9091 9092 9093 9094 9095 9096 9097 9098 9099 9100 9101 9102 9103 9104 9105 9106 9107 9108

do

FILEALL=`ls data/trim/${SMPL}*_art_q20t47_trimf4.fastq`

FILENAME=${FILEALL##*trim/}

FILECORE=${FILENAME%%.fastq}

echo "=================================="

echo "${SMPL}"

echo " mapping..."

bowtie2 -p 6 -x ref/PAC_Ref_01/pac_scaffolds_01 -U ${FILEALL} -S mapping/res/${FILECORE}.sam --sensitive 2> mapping/res/${FILECORE}.stderr

echo " samstat..."

samstat mapping/res/${FILECORE}.sam

echo " SAM > BAM..."

samtools import ref/PAC_Ref_01/pac_scaffolds_01.fa mapping/res/${FILECORE}.sam mapping/res/${FILECORE}.unsorted.bam

samtools sort mapping/res/${FILECORE}.unsorted.bam mapping/res/${FILECORE}.sorted

samtools index mapping/res/${FILECORE}.sorted.bam

echo "=================================="

done

1. **Run bedtools count range**

#! /bin/sh

FILENAME="$1"

BAMPATH="mapping/test_bowtie2/"

if [ "$FILENAME" = "" ]

then

echo "Please provide file name"

echo ""

exit 2

fi

echo "running $FILENAME:"

bedtools multicov -q 15 -bams ${BAMPATH}${FILENAME} -bed mapping/pac_scaffolds_01_genes.gff > ${FILENAME}_q15.tmp

bedtools multicov -q 20 -bams ${BAMPATH}${FILENAME} -bed mapping/pac_scaffolds_01_genes.gff > ${FILENAME}_q20.tmp

bedtools multicov -q 25 -bams ${BAMPATH}${FILENAME} -bed mapping/pac_scaffolds_01_genes.gff > ${FILENAME}_q25.tmp

bedtools multicov -q 30 -bams ${BAMPATH}${FILENAME} -bed mapping/pac_scaffolds_01_genes.gff > ${FILENAME}_q30.tmp

bedtools multicov -q 35 -bams ${BAMPATH}${FILENAME} -bed mapping/pac_scaffolds_01_genes.gff > ${FILENAME}_q35.tmp

bedtools multicov -q 40 -bams ${BAMPATH}${FILENAME} -bed mapping/pac_scaffolds_01_genes.gff > ${FILENAME}_q40.tmp

bedtools multicov -q 45 -bams ${BAMPATH}${FILENAME} -bed mapping/pac_scaffolds_01_genes.gff > ${FILENAME}_q45.tmp

awk '{print $9}' ${FILENAME}_q15.tmp > ${FILENAME}_9_q15.tmp

awk '{print $9}' ${FILENAME}_q20.tmp > ${FILENAME}_9_q20.tmp

awk '{print $9}' ${FILENAME}_q25.tmp > ${FILENAME}_9_q25.tmp

awk '{print $9}' ${FILENAME}_q30.tmp > ${FILENAME}_9_q30.tmp

awk '{print $9}' ${FILENAME}_q35.tmp > ${FILENAME}_9_q35.tmp

awk '{print $9}' ${FILENAME}_q40.tmp > ${FILENAME}_9_q40.tmp

awk '{print $9}' ${FILENAME}_q45.tmp > ${FILENAME}_9_q45.tmp

awk '{print $1"_"$4"_"$5}' ${FILENAME}_q15.tmp > locus.tmp

paste locus.tmp ${FILENAME}_9_q??.tmp > mapping/compare_mapping/${FILENAME}_counts_q15_q45.txt

#rm *.tmp
